# Data Quality and Study Compliance Among College Students Across 2 Recruitment Sources: Two Study Investigation

**DOI:** 10.2196/39488

**Published:** 2022-12-09

**Authors:** Abby L Braitman, Megan Strowger, Jennifer L Shipley, Jordan Ortman, Rachel I MacIntyre, Elizabeth A Bauer

**Affiliations:** 1 Department of Psychology Old Dominion University Norfolk, VA United States; 2 Department of Psychology Millersville University Millersville, PA United States; 3 Department of Psychological and Brain Sciences Texas A&M University College Station, TX United States

**Keywords:** data quality, attention checks, recruitment, retention, college students, mobile phone

## Abstract

**Background:**

Models of satisficing suggest that study participants may not fully process survey items and provide accurate responses when survey burden is higher and when participant motivation is lower. Participants who do not fully process survey instructions can reduce a study’s power and hinder generalizability. Common concerns among researchers using self-report measures are data quality and participant compliance. Similarly, attrition can hurt the power and generalizability of a study.

**Objective:**

Given that college students comprise most samples in psychological studies, especially examinations of student issues and psychological health, it is critical to understand how college student recruitment sources impact data quality (operationalized as attention check items with directive instructions and correct answers) and retention (operationalized as the completion of follow-up surveys over time). This examination aimed to examine the following: whether data quality varies across recruitment sources, whether study retention varies across recruitment sources, the impact of data quality on study variable associations, the impact of data quality on measures of internal consistency, and whether the demographic qualities of participants significantly vary across those who failed attention checks versus those who did not.

**Methods:**

This examination was a follow-up analysis of 2 previously published studies to explore data quality and study compliance. Study 1 was a cross-sectional, web-based survey examining college stressors and psychological health (282/407, 69.3% female; 230/407, 56.5% White, 113/407, 27.8% Black; mean age 22.65, SD 6.73 years). Study 2 was a longitudinal college drinking intervention trial with an in-person baseline session and 2 web-based follow-up surveys (378/528, 71.6% female; 213/528, 40.3% White, 277/528, 52.5% Black; mean age 19.85, SD 1.65 years). Attention checks were included in both studies to assess data quality. Participants for both studies were recruited from a psychology participation pool (a pull-in method; for course credit) and the general student body (a push-out method; for monetary payment or raffle entry).

**Results:**

A greater proportion of participants recruited through the psychology pool failed attention checks in both studies, suggesting poorer data quality. The psychology pool was also associated with lower retention rates over time. After screening out those who failed attention checks, some correlations among the study variables were stronger, some were weaker, and some were fairly similar, potentially suggesting bias introduced by including these participants. Differences among the indicators of internal consistency for the study measures were negligible. Finally, attention check failure was not significantly associated with most demographic characteristics but varied across some racial identities. This suggests that filtering out data from participants who failed attention checks may not limit sample diversity.

**Conclusions:**

Investigators conducting college student research should carefully consider recruitment and include attention checks or other means of detecting poor quality data. Recommendations for researchers are discussed.

## Introduction

### Background

The validity of the findings of any study hinges on the integrity of the data collected. Common concerns among psychology researchers using self-report measures are data quality and participant compliance. Participants may not fully read or process the self-report measure instructions or items, adding noise to data rather than reflecting the constructs being assessed, or they may not complete the study protocol, reducing the number of assessments used for analyses. Both reduce the study’s power [1] and may hinder the generalizability of its findings [2,3]. Given that clinical trials of psychological treatments are chronically underpowered [4], reduced power due to poor data quality can exacerbate the lack of trust in the study findings. To prevent these negative effects on statistical power and external validity, researchers may aim to recruit compliant participants, better incentivize compliance, and detect and remove noncompliant participants from data sets. Given that college students comprise most samples in psychological studies [5-7], it is necessary to understand the associations between recruitment sources targeting college students and participant compliance. For this paper, we defined participant compliance as providing high quality data (ie, putting in reasonable effort and fully reading each item before responding) and completing follow-up assessments (ie, retention; only applicable for longitudinal studies).

One approach to identifying participant noncompliance that affects data quality is to use attention check items with instructions to select a particular answer (eg, “Select ‘slightly agree’ for this item”) or that have factual answers (eg, “Which number is largest?”); these are also called instructional manipulation checks [1], bogus items [8], infrequency scales [9], or random-responding indicators [10]. These items can identify participants who are satisficing (ie, putting in minimal effort and potentially not fully reading or comprehending each item), which is sometimes called careless responding. Removing these participants may increase statistical power such that correlations are stronger among relevant study variables and experimental effects across conditions are larger [1,10-12] or may otherwise reduce “noise” among study variable associations [13]. Identifying poor quality data where responses may not reflect the true study construct via attention checks and removing these satisficing cases may reduce random error and increase statistical power, but it also may result in removing a meaningful group and introducing bias, as certain demographics (eg, gender, age, race, education, and intrinsic motivation) can be associated with satisficing [11,14]. It is possible that some recruitment sources may yield participants who are not only less inclined to satisfice but are also more demographically diverse, allowing for the removal of satisficing participants without limiting the diversity and generalizability of the sample.

In a model explaining how individuals formulate and respond to survey questions, Tourangeau et al [15] proposed that poor data quality comes from failing to engage in at least one of the four stages of the cognitive processing: (1) understanding the meaning of the item, (2) finding information in memory relevant to the item, (3) summarizing the information found, and (4) using that information to choose a response given the options. Satisficing could result from failing to engage in any one of these stages of cognitive processing. However, the attention checks used in this examination as an indicator of data quality are designed to detect the most egregious forms of satisficing (stage 1: not fully reading and understanding the item) rather than the later forms of cognitive processing (eg, taking the time to process exactly how anxious they remember being or whether this is best reflected as an endorsement of 4 vs 5 for a given item). The study by Krosnick [16] gave an overview of a variety of response strategies that respondents may choose when engaging in satisficing to conserve their mental energy and suggested that some individuals may choose to engage in satisficing to conserve their mental energy when faced with great task difficulty (eg, many items in a survey) and that the likelihood of satisficing increases as the burden of the survey increases, and participants become less motivated to perform well as they become more fatigued. Moreover, it suggested that respondents may first be less diligent about the later stages of cognitive processing (eg, taking the time to decide between 4 and 5) before fully omitting stages (eg, not fully reading the item). A systematic review of 141 studies that included various indicators of satisficing revealed that 74% of the studies found that task difficulty was significantly associated with satisficing, and 68% of the studies found that respondent motivation was significantly associated with satisficing [17], suggesting that satisficing is a result of the qualities of both the survey task (highly burdensome) and the participant (low motivation).

Another issue of participant compliance particularly important for longitudinal research such as examinations of psychological health is study retention rates. Many studies require multiple assessments, such as observing natural developmental trajectories or following changes in behaviors, symptoms, or attitudes after the administration of an intervention. Although interventions offer additional benefits to participants compared with nonintervention research (eg, potential improvements in mental or physical health), these benefits are often obtained immediately and do not extend to incentivizing retention for follow-up surveys. Moreover, follow-up assessments are typically administered remotely and after substantial time has passed (eg, weeks or months). Therefore, retention rates typically drop with each additional follow-up. For example, challenges with retention have been noted in a meta-analysis of cohort studies on mental and behavioral health [18], a meta-analysis of dissonance-based interventions for health behavior change [19], and a meta-analysis of digital interventions for the treatment and prevention of eating disorders [20]. This is particularly challenging for studies involving college students. An integrative analysis of 24 studies of brief interventions for college drinking found retention rates as low as 46% for the 6-month follow-ups and 51% for the 9- to 12-month follow-ups [21]. The authors of these studies have noted how challenging it can be to retain college students, particularly when administering interventions such as those for college drinking [22,23] or web-based programs for students with depression, anxiety, or stress [24]. Identifying recruitment methods that are associated with better data quality and higher compliance (eg, passing attention checks and completing study protocols including follow-up assessments) may reduce the costs associated with longitudinal research, increase the benefits and practicality of the study designs, and strengthen the trust in the study findings.

### Recruitment Sources

Undergraduate college students serve as study samples in most psychology studies, a consistent trend over time. An investigation of 6 top journals in multiple fields of psychology over 20 years (1975, 1985, and 1995) included 1559 articles with human participants [7]. Researchers found that most studies (68%) exclusively used undergraduate college student samples and that this finding was consistent over time (69.8% in 1975, 66.7% in 1985, and 68.2% in 1995). An investigation into 1 specific premier journal, the *Journal of Personality and Social Psychology*, revealed that 67% of American study samples were specifically undergraduate students enrolled in psychology courses, and this rose to 80% of samples for non-American studies [5]; however, this number has dropped to 42% overall (39% of American-based studies and 54% of non-American studies) in more recent years [6]. This makes it imperative to examine study compliance among college students and, in particular, whether the use of psychology student pools has an impact.

Student participant pools have received both praise and criticism from the psychology community [25,26]. They provide researchers with a low-cost and efficient recruitment source, which may be particularly important for student researchers who do not have funding [26]. Although there are concerns that student participant pools mainly comprise female, White, and young psychology majors [26,27] and that this can result in samples that do not generalize beyond Western, educated, industrialized, rich, and democratic societies [28], student participation pools are becoming more demographically diverse, mirroring the increasing diversity among those attending college [26]. Moreover, some research questions focus specifically on student populations (eg, studies that focus on unique college stressors and their links to mental health or interventions targeting college drinking), creating a need for student participant sources.

Student participation pools may potentially reflect the true population of interest in addition to being convenient; however, these pools can also be associated with lower enrollment and study compliance. Sharpe and Poets [26] found that as many as 56.7% of students in 2 large introductory courses chose not to participate in research or earn any research credits. Motivational issues and time commitment are the 2 primary factors linked to student nonparticipation in research pools [29,30]. It is possible that factors contributing to low motivation to participate may also impact study compliance among those who choose to participate.

Antoun et al [31] suggested that the type of recruitment method may explain the differences in data quality, differentiating between pull-in and push-out recruitment. They defined pull-in recruitment methods as those that post studies to participant pools already opting into research to some degree, such as Amazon Mechanical Turk (MTurk) workers or individuals looking for paid research opportunities on Craigslist (similar to student participant pools at academic institutions), whereas push-out recruitment uses methods in which advertisements are posted in venues not already focused on research, such as advertisements on websites not dedicated to the purpose of recruiting study participants (eg, Facebook advertisements), flyers, and email blasts. In a study comparing pull-in versus push-out approaches to recruit iPhone users for a cross-sectional web-based survey, Antoun et al [31] found that pull-in methods (using Craigslist and MTurk) were more efficient in recruiting participants, in that the rate of enrollment was faster and the cost per participant who enrolled in the study was lower, than push-out methods (using paid advertisements on Google and Facebook). Although no attention checks were included in the data collection, the authors concluded that the participants recruited through pull-in methods provided better data (ie, fewer “don’t know” responses and fewer skipped or incomplete responses), possibly indicating less satisficing. Multiple studies have extended these findings by recruiting samples across both pull-in and push-out approaches and including attention checks as indicators of data quality. One such study recruited participants from MTurk (pull-in), Facebook (push-out), and Qualtrics panels (pull-in) and included 1 attention check [32]. They found that the rate of passing the attention check question was highest among the participants recruited through MTurk (93%) compared with those recruited through Facebook advertisements (66%) and Qualtrics panels (40%). The participants recruited through MTurk endorsed “don’t know” responses only 0.4% of the time compared with those recruited through Facebook (4%) and Qualtrics panels (5%). These findings suggested that the push-out and pull-in distinction may be less important than the sources, given that both the highest (MTurk) and lowest (Qualtrics panels) rates of attention check failure were associated with pull-in sources. A similar study found that MTurk samples were more likely to pass the attention check (97.5%) than panel respondents via Dynata (91.6%), which were both pull-in sources [33]. These findings suggested that further research into the pull-in versus push-out distinction is necessary; differences in participant compliance have not been explored using the pull-in and push-out recruitment methods more commonly used on college campuses (ie, pull-in: a psychology student participation pool; push-out: email announcements to the general undergraduate student body). Moreover, no study to date has explored study compliance across these 2 sources among college students.

### This Examination: A Two Study Investigation

This examination explored study compliance (ie, data quality and retention) by recruitment source across 2 studies of college students in the United States with varying design protocols. These were follow-up analyses of published studies with different primary research goals. Study 1 focused on unique college stressors and links to mental health [34] and involved remotely distributing a web-based survey (fully remote and cross-sectional design). Study 2 examined an intervention targeting college drinking (ClinicalTrials.gov NCT03440463) [35] and involved an in-person baseline procedure with a computerized survey and remote web-based follow-up surveys 1 month and 3 months later (in-person component and longitudinal design). For both studies, participants were recruited from (1) a psychology student participation pool, receiving research credit in psychology courses as compensation and (2) the general student body via emailed announcements, receiving either a raffle entry (study 1) or monetary compensation (study 2).

This examination aimed to examine the following: (1) whether data quality varied across recruitment sources, (2) whether study retention varied across recruitment sources, (3) the impact of data quality on study variable associations, (4) the impact of data quality on measures of internal consistency, and (5) whether the demographic qualities of participants significantly varied across those who failed attention checks versus those who did not. Data quality was examined with attention checks that were used in both study 1 and study 2, and retention was operationalized as follow-up completion rates in study 2 only. Given the limited research on study compliance by recruitment methods with college samples, the analyses for aims 1, 2, and 5 were exploratory in nature. For aims 3 and 4, consistent with previous findings that satisficing can add noise to the assessment and reduce the strength of effects [1,11], we hypothesized that both internal consistency indicators and study variable associations would be stronger after eliminating those who failed attention checks. In particular, satisficing participants tend to endorse midpoints across multiple measures [10], potentially reducing the strength of association among variables, and the inclusion of satisficing participants can mask the strong effects that are revealed after their removal [1], supporting our hypothesis for aim 3. In addition to tendencies to endorse scale midpoints, satisficing participants also fail to notice scale reversals (ie, reverse-scored items) [11], potentially reducing indicators of internal consistency, supporting our hypothesis for aim 4.

## Study 1

### Methods

Study 1 was a cross-sectional examination of worry as a mediator between psychosocial stressors and anxiety, stress, and depression [34]. The main outcomes of interest to the original study included worry, stress, depression, and anxiety.

#### Participants

Undergraduate students (282/407, 69.3% female; 230/407, 56.5% White; mean age 22.65, SD 6.73 years) from a large, public, minority-serving university in the mid-Atlantic region of the United States were recruited via university-wide student announcements (a push-out approach; n=257) as well as through the psychology student research pool (a pull-in approach; n=150) to complete a web-based survey. They were relatively evenly distributed across the year in school. Refer to Table 1 for relevant demographic information for the full sample as well as categorized based on recruitment source. Both recruitment advertisements mentioned that the study was a web-based survey and the type of information assessed (eg, anxiety, worry, and related cognitions). Both indicated an estimate of how long the survey would take and information about compensation. Only the university-wide student announcement included a sentence about how the data would be used and that their data would remain confidential, as that detail was already clear for participants from the psychology pool. Different links were provided for recruitment through the psychology pool versus university-wide announcements. The 2 data sets were coded to reflect how participants accessed the survey and then merged.

**Table 1:** Descriptive information of the study 1 sample categorized by recruitment source^a^.

Variable		General student announcements (n=257)	Psychology pool (n=150)	Total (N=407)	*P* value	
**Gender, n (%)**						.32
	Female	168 (75.7)	114 (76.5)	282 (69.3)		
	Male	47 (21.2)	34 (22.8)	81 (19.9)		
	Transgender	5 (2.3)	0 (0)	5 (1.2)		
	Other	2 (0.9)	1 (0.7)	3 (0.9)		
**Ethnicity, n (%)**						.24
	Hispanic or Latinx	16 (7.2)	16 (10.7)	32 (8.6)		
	Not Hispanic or Latinx	205 (92.8)	133 (89.3)	338 (91.4)		
**Race^b^, n (%)**						
	Asian	32 (12.5)	15 (10)	47 (11.5)	.46	
	Black or African American	54 (21)	59 (39.3)	113 (27.8)	*<.001* ^c^	
	Native American	7 (2.7)	3 (2)	10 (2.5)	.75	
	Other	10 (3.9)	7 (4.7)	17 (4.2)	.71	
	White	146 (56.8)	84 (56)	230 (56.5)	.87	
**Year in school, n (%)**						*.001*
	Freshman	53 (24)	65 (43.6)	118 (31.9)		
	Sophomore	44 (19.9)	21 (14.1)	65 (17.6)		
	Junior	56 (25.3)	27 (18.1)	83 (22.4)		
	Senior	68 (30.8)	36 (24.2)	104 (28.1)		
**Employment, n (%)**						*.02*
	Employed	134 (60.6)	68 (45.6)	202 (54.6)		
	Not employed	82 (37.1)	71 (47.7)	153 (41.4)		
	Other	5 (2.3)	10 (6.7)	15 (3.7)		
Age (years), mean (SD)		22.71 (6.19)	22.58 (7.48)	22.65 (6.73)	.86	

^a^Categories with <5 participants per cell were not included in the chi-square examinations.

^b^The participants could select >1 response option for race, so tallies may sum up to more than the total sample size.

^c^Significant *P* values are indicated in italics.

#### Procedure

A study advertisement was included in the student announcements emailed to all the students at the host institution. Interested students could click on a link to complete the web-based survey. A similar advertisement was included in the web-based portal for the psychology research pool, which was linked to the same survey. The psychology participation pool included students enrolled in psychology courses. In exchange for their participation in the studies posted, they were provided research credit that they can apply to a course in which they were enrolled. Instructors may build these credits into the grading criteria for the course or offer the students extra credit. The students might sign up for any study for which they were eligible. Volunteering as a study participant was not required to earn these research credits; students might alternatively complete scientific article critiques. Student announcements were emailed to every student enrolled in the university each day. They included announcements for academic workshops, research studies, social activities, and employment opportunities available to the students. Data collection for study 1 took place from July to September 2017.

#### Ethical Considerations

We complied with American Psychological Association’s ethical standards in the treatment of our sample. The Human Subjects Review Committee of the institution determined the study to be exempt from ongoing oversight (reference number 1103992-1). All participants provided informed consent before completing the survey. The participants recruited via student announcements had the choice of being compensated with either a raffle entry (one of 4 US $50 Amazon gift cards or one of 12 US $25 Amazon gift cards) or research credit (if applicable). Participants recruited via the psychology student research pool were compensated with research credit. Because information for compensation purposes was collected in a separate questionnaire not linked to survey responses, the study data were anonymous.

#### Materials

##### Worry

Worry was assessed using the Penn State Worry Questionnaire [36], which is a 16-item measure that assesses the severity of participants’ worries (eg, my worries overwhelm me). Response options ranged from 1=*not at all typical of me* to 5=*very typical of me*.

##### Stress, Depression, and Anxiety

Stress, depression, and anxiety were assessed with the 21-item Depression Anxiety Stress Scale [37]. Each construct was assessed with 7 items, including stress (eg, I found myself getting upset rather easily), depression (eg, I felt that life was meaningless), and anxiety (eg, I felt I was close to panic). Responses ranged from 0=*did not apply to me at all over the last week* to 3=*applied to me very much or most of the time over the past week*.

##### Attention Checks

In total, 8 attention check questions were added to the surveys to assess data quality or, more specifically, to detect satisficing, where inattentive participants were not fully reading survey items or instructions. Of these, 4 were separate questions (eg, Select the highest number), and 4 were integrated into questionnaires (eg, Select “5-7 days” for this answer). The number of incorrect responses was summed and then recoded into a series of variables that represented whether the participants answered any of the attention checks incorrectly (n=55), ≥2 incorrectly (n=16), or ≥3 incorrectly (n=9; 0=*no* and 1=*yes* for all variables). A variable was not created for answering ≥4 incorrectly, as this represented only 1 participant.

#### Analysis Approach

The demographic characteristics of the sample were compared across recruitment sources using chi-square tests for categorical variables (eg, year in school and employment) and 2-tailed *t* tests for continuous variables (ie, age). To test study aim 1 (data quality across recruitment sources), the proportion of participants failing attention checks (coded for failing ≥1, ≥2, or ≥3 as *yes* vs *no*) was compared against the recruitment source (general student body vs psychology pool) using a series of chi-square tests of independence (or Fisher exact when the expected value for any cell was <5). This examination was repeated as a series of logistic regressions to control for any demographic characteristics that significantly varied across recruitment sources. We considered the survey completion time as another marker of data quality. However, time spent on the survey could be impacted by multiple factors, such as satisficing (potentially resulting in faster completion times than other participants) or distraction (potentially resulting in slower completion times than other participants). However, survey completion time could also be impacted by external factors not related to the quality of the data such as poor internet connection or taking a break and coming back, which would result in slower completion times, but responses may still be of high quality. It could also be impacted by skipping some items or not completing the full survey, which would result in faster completion times, but the completed responses may still be of high quality. Moreover, if researchers use fast survey completion times to throw out cases, throwing out those who only complete part of the survey, they could introduce systematic bias by using a complete-case analysis, which is labeled as one of the worst methods for addressing missing data by the American Psychological Association Task Force on Statistical Inference [38]. As such, we chose to focus exclusively on failed attention checks as a marker of poor data quality. For the same reason, we chose to focus on failing attention checks (ie, completing the item but getting it wrong) as opposed to answering the item correctly, as this approach allowed the participants to drop out of the survey and not complete all attention check items while still potentially providing good quality data for the items answered.

Aim 2 (whether study retention varied across recruitment sources) was not examined for study 1 because it was not longitudinal. To test study aim 3 (the impact of data quality on study variable associations), a series of bivariate correlations were conducted among the variables of interest to the original study (ie, worry, stress, depression, and anxiety). These were conducted once for the full sample and then again for only those who did not fail any attention checks, those who failed ≤1 attention checks, those who failed ≤2 attention checks, and those who failed ≤3 attention checks (ie, retaining those who were not *engaging* in satisficing using various cutoffs). Finally, they were conducted again for those who failed at least one attention check (ie, among those who were *engaging* in satisficing). Correlations were not conducted among those who failed ≥2 (or ≥3) attention checks because of the small number of participants meeting these criteria (ie, ≤16). The largest discrepancies between the correlations for those who failed any attention checks and those who did not were examined via Fisher *z* for independent samples. The comparisons were only conducted for those who failed any attention checks versus none, as they represented a split of the full sample (ie, none of the participants were in both groups). This allowed us to detect whether significant noise was introduced to the sample by those engaging in satisficing, potentially reducing the strength of associations or increasing SEs via random error.

To examine aim 4 (the impact of data quality on measures of internal consistency), Cronbach α and McDonald omega were calculated for the key study measures using the full sample and then again only for those who did or did not fail varying numbers of attention checks. Both indicators were provided because McDonald omega has more realistic and attainable assumptions and thus may be a more accurate indicator of internal consistency in many circumstances, but Cronbach α is more widely used and understood [39]. Finally, a series of 2-tailed *t* tests and chi-square tests were conducted to test study aim 5, which is to explore whether the demographic qualities of participants significantly varied across those who failed any attention checks versus those who did not. All analyses were conducted using SPSS statistical software (version 26; IBM Corp; (including using a macro by Hayes and Coutts [39] for McDonald omega). Sample size for the original examination [34] was determined via a power analysis using G*Power [40], specifying a 2-tailed test, an α of .05, and a power of 0.80. Power analysis was not repeated for this study because it was a secondary analysis.

### Results

#### Overview

As shown in Table 1, significantly more Black or African American students were recruited through the psychology pool (59/150, 39.3%) than through the general student body (54/257, 21%; *P*<.001). In addition, the sample recruited via the psychology pool had significantly more first-year students (almost a majority; 65/150, 43.6%), whereas the sample recruited via the general student body was generally more balanced across years in school (*P*=.001). Finally, significantly more participants recruited via the general student body were employed (134/257, 60.6%) than those from the psychology pool (68/150, 45.6%; *P*=.02). The sample did not significantly vary across recruitment methods for gender, age, ethnicity, or other racial identities.

#### Aim 1: Data Quality by Recruitment Source

Across the total sample, 86.5% (352/407) of the participants did not fail any attention checks, 9.6% (39/407) failed 1 check, 1.7% (7/407) failed 2 checks, 2% (8/407) failed 3 checks, and 0.2% (1/407) failed 5 checks. No one failed >5 (out of 8) checks. The recruitment method type was associated with data quality such that more psychology pool participants (29/150, 19.3%) failed any attention checks than the general student body participants (26/257, 10.1%; *χ^2^*_1_=6.9, *P*=.009). Similarly, more psychology pool participants (10/150, 6.7%) failed ≥2 attention checks than the general student body participants (6/257, 2.3%; *χ^2^*_1_=4.7, *P*=.03). Although the trend was in the same direction for failing ≥3 attention checks (6/150, 4% vs 3/257, 1.2% for psychology pool participants compared with the general student body participants), this did not reach statistical significance based on Fisher exact test (*P*=.08).

These comparisons were repeated as a series of logistic regressions, controlling for the demographics that were significantly different across recruitment sources (year in school, employment, and endorsing Black or African American for race). Year in school and employment did not significantly predict attention check failure and were dropped as predictors. The model controlling for the endorsement of Black or African American identity was consistent with the chi-square analysis, finding that the recruitment source was significantly associated with any attention check failure, with the participants recruited via the psychology pool significantly more likely to fail the attention checks (B=0.63; *P*=.04; exp[B]=1.87, 95% CI 1.04-3.37). Controlling for race, the recruitment source was not significantly associated with failing ≥2 attention checks (B=0.83; *P*=.13; exp[B]=2.28, 95% CI 0.79-6.60), or ≥3 attention checks (B=0.76; *P*=.30; exp[B]=2.13. 95% CI 0.51-8.99).

#### Aim 3: Impact of Data Quality on Variable Associations

Correlations among the key variables for the original study (ie, worry, stress, depression, and anxiety) were conducted for the full sample, those who did not fail any attention checks, those who had failed at least one attention check, those who failed <2, and those who failed <3 attention checks (Table 2). Before the analysis, the variables were examined for extreme values (ie, outliers) and normality. All variables were found to be normally distributed, and no extreme outliers were identified. Overall, when comparing the full sample with those who did not fail any attention checks, there was no clear pattern of differences; some correlations became smaller, whereas others were larger. Similarly, there were mixed findings when comparing the strength of correlations between the participants who did not fail any attention checks and those who did. As expected, correlations for those who failed <2 or <3 attention checks were midrange between those who did not fail any and those who failed at least one.

The changes in correlations between those who did not fail any attention checks and those who did were compared with Fisher *z* independent sample comparisons to examine the magnitude of difference. Contrary to what was hypothesized, the association between depression and anxiety was significantly stronger among the participants who failed at least one attention check than among those who did not fail any (*z* score −3.11; *P*=.001), as was the association between stress and anxiety (*z* score −3.66; *P*<.001). The next largest differences (between stress and worry: *z*=1.60; *P*=.06 and between anxiety and worry: *z*=1.54; *P*=.06) were in the expected direction but were not significant. The differences between all other correlations were smaller in magnitude and were not significantly different across groups by attention check failure.

**Table 2:** Correlations among key study 1 variables categorized based on attention check failure^a^.

Measure		1	2	3	4
**Full sample (** **N** **=407)**					
	1. Worry	—^b^	—	—	—
	2. Stress	.62^a^	—	—	—
	3. Depression	.49^a^	.73^a^	—	—
	4. Anxiety	.57^a^	.79^a^	.69^a^	—
**Did not fail any attention checks (n=352)**					
	1. Worry	—	—	—	—
	2. Stress	.64^a^	—	—	—
	3. Depression	.51^a^	.72^a^	—	—
	4. Anxiety	.60^a^	.77^a^	.66^a^	—
**Failed >1 attention checks (n=54)**					
	1. Worry	—	—	—	—
	2. Stress	.47	—	—	—
	3. Depression	.43	.81	—	—
	4. Anxiety	.43	.92	.85	—
**Failed <2 attention checks (n=391)**					
	1. Worry	—	—	—	—
	2. Stress	.62	—	—	—
	3. Depression	.50	.72	—	—
	4. Anxiety	.59	.78	.68	—
**Failed <3 attention checks (n=394)**					
	1. Worry	—	—	—	—
	2. Stress	.63	—	—	—
	3. Depression	.51	.73	—	—
	4. Anxiety	.58	.79	.68	—

^a^All correlations were significant at *P*<.001.

^b^Not applicable.

#### Aim 4: Impact of Data Quality on Internal Consistency

As shown in Table 3, differences in Cronbach α and McDonald omega were negligible across samples restricted in size by attention check failure.

**Table 3:** Internal consistency measures among key study 1 variables by attention check failure.

Variables	No attention failures (n=352)		No more than 1 failure (n=391)		No more than 2 failures (n=398)			No more than 3 failures (n=406)			Full sample (N=407)	
	α	Ω	α	Ω	α	Ω	α		Ω	α		Ω
Worry	.935	0.941	.933	0.940	.933	0.939	.932		0.938	.931		0.938
Stress	.865	.0868	.868	0.870	.871	0.873	.872		0.874	.872		0.874
Anxiety	.853	0.856	.859	0.861	.861	0.863	.863		0.865	.862		0.865
Depression	.905	0.907	.912	0.914	.913	0.914	.912		0.913	.912		0.913

#### Aim 5: Demographics by Attention Check Failure

A series of chi-square analyses revealed that attention check failure was not significantly associated with gender (*χ^2^*_3_=1.7, *P*=.63), ethnicity (*χ^2^*_1_=0.0, *P*=.86), year in school (*χ^2^*_3_=0.9, *P*=.81), employment status (*χ^2^*_4_=4.2, *P*=.38), or age (t_365_=0.68; *P*=.50). Although it was not associated with the endorsement of some racial identities (ie, identifying as Asian: *χ^2^*_1_=0.09, *P*=.77 or Native American: *χ^2^*_1_=0.4, *P*=.54), it was significantly associated with identifying as Black or African American (*χ^2^*_1_=7.9, *P*=.005) and as White (*χ^2^*_1_=10.5, *P*=.001). More participants who identified as Black or African American failed at least one attention check (21.2%) than those who did not identify as Black (10.5%), whereas fewer participants who identified as White failed the attention checks (8.7%) than those who did not identify as White (19.8%). Given the different demographic breakdown by recruitment source, we also examined attention check failure and race within recruitment source. Identifying as Black was still significantly associated with attention check failure within the psychology pool (*χ^2^*_1_=5.3, *P*=.03). A similar trend was observed for the announcement pool (*χ^2^*_1_=1.0, *P*=.31), but it was not significant (likely because of the smaller sample size).

Comparing failing ≥2 attention checks with failing <2 checks revealed the same general pattern of findings. There were no significant associations between attention check failure and most demographic variables. However, more participants who identified as Black or African American failed ≥2 attention checks (8.8%) than those who did not identify as Black (2%; Fisher exact *P*=.003), whereas fewer participants who identified as White failed ≥2 attention checks (1.7%) than those who did not identify as White (6.8%; *χ^2^*_1_=6.7, *P*=.009).

Comparing failing ≥3 attention checks with failing <3 checks also revealed the same general pattern of findings. There were no significant associations between attention check failure and most demographic variables. However, more participants who identified as Black or African American failed ≥2 attention checks (7.1%) than participants who did not identify as Black (0.3%; Fisher exact *P*<.001), whereas fewer participants who identified as White failed ≥2 attention checks (0%) than those who did not identify as White (5.1%; Fisher exact *P*<.001).

## Study 2

### Methods

Study 2 was a longitudinal (ClinicalTrials.gov NCT03440463) randomized control trial designed to examine the effects on drinking outcomes of personalized normative feedback booster emails sent after completing a web-based alcohol intervention administered in person [35]. The main outcomes of interest to the original study included alcohol consumption, alcohol-related problems, and descriptive normative perceptions (ie, how much one thinks relevant others drink).

#### Participants

Participants (378/528, 71.6% female; 281/528, 53.2% Black or African American; 215/528, 40.9% White; mean age 19.85 years, SD 1.65 years) were recruited from the same university as the one from where study 1 participants were recruited (a large, public, minority-serving institution in the mid-Atlantic region of the United States) through 2 recruitment sources: via student announcement emails (a push-out approach; n=127) and a psychology research pool (a pull-in approach; n=401). Eligible participants were current students aged between 18 and 24 years who had consumed at least one alcoholic beverage in the past 2 weeks. Refer to Table 4 for relevant demographic information for the full sample as well as categorized by recruitment source. Both recruitment advertisements mentioned that the study required in-person attendance for the first session and that it investigated the effects of a computerized intervention on student health behaviors, such as drinking, over an extended period. Both indicated eligibility criteria, an estimate of how long the first session would take, and information about compensation. Only the university-wide student announcement mentioned that the data would remain confidential, as that detail was already clear for participants from the psychology pool.

**Table 4:** Study 2 sample descriptive information categorized by recruitment source^a^.

Variable			General student announcements (n=127)		Psychology pool (n=401)	Total (N=528)		*P* value	
**Gender, n (%)**									.85
	Female	91 (71.7)		287 (71.6)		378 (71.6)			
	Male	36 (28.3)		113 (28.2)		149 (28.2)			
	Transgender	0 (0)		0 (0)		0 9 (0)			
	Other	0 (0)		1 (0.2)		1 (0.2)			
**Ethnicity, n (%)**									.75
	Hispanic or Latinx	15 (11.8)		43 (10.8)		58 (11)			
	Not Hispanic or Latinx	112 (88.2)		355 (89.2)		467 (89)			
**Race^b^, n (%)**									
	Asian	12 (9.4)		39 (9.9)		51 (9.8)	.88		
	Black or African American	64 (50.4)		213 (54.1)		277 (53.2)	.47		
	Native American	8 (6.3)		11 (2.8)		19 (3.6)	.07		
	Other	12 (9.4)		24 (6.1)		36 (6.9)	.19		
	White	49 (38.6)		164 (41.6)		213 (40.9)	.54		
**Year in school** **, n (%)**									*<.001* ^c^
	Freshman	21 (16.5)		156 (38.9)		177 (33.5)			
	Sophomore	27 (21.3)		123 (30.7)		150 (28.4)			
	Junior	32 (25.2)		64 (16)		96 (18.2)			
	Senior	46 (36.2)		56 (14)		102 (19.3)			
	Graduate	0 (0)		1 (0.2)		1 (0.2)			
	Other	1 (0.8)		1 (0.2)		2 (0.4)			
Age (years), mean (SD)			20.51 (1.66)		19.65 (1.60)	19.85 (1.65)		*<.001*	

^a^Categories with <5 participants per cell were not included in the chi-square examinations.

^b^Participants could select >1 response option for race, so tallies may sum up to more than the sample size.

^c^Significant *P* values indicated in italics.

#### Procedure

A study advertisement was included in the emailed student announcements sent to all the students at the host institution. Interested students could click on a link to complete a screener survey. Eligible individuals were directed to a web-based scheduler to select an upcoming appointment. A similar advertisement was included in the web-based portal for the psychology research pool. The structure of the psychology participation pool was identical to that of the first study; students enrolled in psychology courses could earn research credit in exchange for their participation in the studies posted or by writing scientific article critiques. The portal allowed for the advertisement to be viewed only by students who met the restricted age criterion. The psychology pool participants did not need to complete the screener survey (the alcohol criterion was prominently displayed in the study description) and could immediately access a web-based scheduler to select an upcoming appointment. All the participants were informed that participating in the study involved attending a 90-minute time slot at the research laboratory where they would be instructed to complete a web-based survey (information of the nature of the constructs were provided) and an alcohol intervention. The participants attended their baseline session in an on-campus research laboratory. After providing informed consent, they completed the baseline survey before completing the web-based alcohol intervention. Participants were randomized into 3 conditions that varied based on the feedback they were provided 2 weeks later via email. All the participants received follow-up surveys via emailed invitations 1 month and 3 months after baseline; those who opted in received reminders via text messages as well. These follow-up surveys were shorter than the initial baseline survey and were completed on the web, so the participants did not have to return to the research laboratory. Baseline data were collected from April 2017 to December 2017.

#### Ethical Considerations

We complied with American Psychological Association's ethical standards in the treatment of our sample. The Old Dominion University Institutional Review Board approved the study (reference number 690348-2). The participants provided informed consent before beginning the survey during the baseline session. The participants who were recruited through the psychology research pool could choose to earn research credit or monetary compensation (US $20) for completing the baseline survey. Participants who were recruited through the general student body received monetary compensation (US $20) for completing the baseline survey. All the participants received monetary compensation for completing the follow-up surveys (US $10 each) and a bonus (US $10) for completing both follow-up surveys. To ensure confidentiality, after data collection and cleaning were complete, all data were deidentified.

#### Materials

##### Drinking Outcomes

The Daily Drinking Questionnaire [41] was used to assess alcohol consumption during each day of a typical week in the past 30 days. The participants were asked to enter the total number of standard drinks consumed on each day of the week as well as the number of hours that passed while they were drinking on those days. Typical drinks per drinking day were calculated by dividing the typical quantity of drinks consumed per week by the total number of drinking days per week. Typical estimated blood alcohol concentration (eBAC) was calculated by averaging the eBAC levels for each drinking day. These levels were calculated based on the number of drinks consumed, hours passed while drinking, and body composition based on sex and weight [42].

##### Descriptive Norms

The Daily Drinking Questionnaire [41] was modified so that participants reported how many standard drinks they believe their close friends consume on each day of a typical week. Descriptive norms reflecting perceived drinks per drinking day for their close friends were calculated by dividing the total number of drinks in a typical week by the number of drinking days.

##### Alcohol-Related Problems

The Young Adult Alcohol Consequences Questionnaire [43] was used to measure the total number of consequences a participant reported for the past 30 days. A total of 48 items assessed consequences across 8 domains (eg, impaired control, academic or occupational consequences, and social or interpersonal consequences). The participants reported whether they experienced the consequence (*yes*) or not (*no*); the number of reported consequences were summed.

##### Attention Checks

In total, 4 attention check questions were added to the surveys to assess data quality or, more specifically, to detect satisficing, where inattentive participants were not fully reading survey items or instructions. Of these questions, 2 were separate questions (eg, Which is the highest number?) and 2 were integrated into questionnaires (eg, Select “Neutral” for this question). The number of incorrect responses was summed and then recoded into a variable that represented whether the participants answered any of the attention checks incorrectly (n=64) or ≥2 incorrectly (n=16; 0=*no* and 1=*yes* for all variables). A variable was not created for answering ≥3 questions incorrectly, as this represented only 3 participants.

#### Analysis Approach

As with study 1, the demographic characteristics of the sample were compared across recruitment sources using chi-square tests for categorical variables (eg, year in school and employment) and 2-tailed *t* tests for continuous variables (ie, age). To test study aim 1 (data quality across recruitment sources), the proportion of participants failing attention checks (*yes* vs *no*) was compared against recruitment source (psychology pool vs general student body) using 3 chi-square tests of independence (1 for each wave of data collection). This examination was repeated as a series of logistic regressions to control for any demographic characteristics that significantly varied across recruitment sources.

To test study aim 2 (study compliance or retention across recruitment sources), the proportion of participants who completed each follow-up survey (*yes* vs *no*) was compared against the recruitment source using 2 chi-square tests of independence (1 for each follow-up survey). These comparisons were also repeated as a pair of logistic regressions to control for any demographic characteristics that significantly varied across recruitment sources. To test study aim 3 (the impact of data quality on study variable associations), a series of bivariate correlations were conducted among the variables of interest to the original study (ie, typical alcohol consumption, typical eBAC, alcohol-related problems, and descriptive norms). These were conducted once for the full sample, then again for only those who did not fail any attention checks, a third time only for those who failed at least one attention check, and, finally, a fourth time for those who failed <2 attention checks. Correlations were not conducted among those who failed ≥2 attention checks because of the small number of participants meeting this criterion (ie, ≤16). The largest discrepancies between the correlations for those who failed attention checks and those who did not were examined using Fisher *z* for independent samples.

To examine aim 4 (the impact of data quality on internal consistency indicators), Cronbach α and McDonald omega were calculated for the only traditional measure (alcohol-related problems) using the full sample and then again only for those who did or did not fail varying numbers of attention checks. Finally, to test study aim 5, a series of 2-tailed *t* tests and chi-square tests were conducted to explore whether the demographic qualities of participants significantly varied across those who failed any attention checks versus those who did not. All analyses were conducted using SPSS (version 26; IBM) (including using a macro by Hayes and Coutts [39] for McDonald omega). The sample size for the original examination [35] was determined via a power analysis using Monte Carlo simulation methods, specifying a 2-tailed test, an α of .05, and a power of 0.80. Power analysis was not repeated for this study because it was a secondary analysis.

### Results

#### Overview

As shown in Table 4, the sample recruited via the psychology pool had significantly more freshmen (156/401, 38.9%) and sophomore (123/401, 30.7%) students than upper classmen, whereas the sample recruited via the general student body was generally more balanced across year in school (*P*<.001). The psychology pool participants were also slightly younger (mean age 19.65, SD 1.60 years) than the participants recruited via the general student body (mean age 20.51 years, SD 1.66 years; *P*<.001). The sample did not significantly vary across recruitment methods for gender, ethnicity, or race.

#### Aim 1: Data Quality by Recruitment Source

Across the total sample for study 2, at baseline, 87.9% (464/528) of the participants did not fail any attention checks, 9.1% (48/528) failed 1 check, 2.5% (13/528) failed 2 checks, and 0.6% (3/528) failed 3 checks. No one failed 4 attention checks. Recruitment type was associated with data quality for the baseline protocol (*χ^2^*_1_=4.0, *P*=.046), with more psychology pool participants (55/401, 13.7%) failing any attention checks than those from the general student body (9/127, 7.1%). Failing ≥2 attention checks was not significantly different between the psychology pool participants (15/401, 3.7%) and those from the general student body (1/127, 0.8%; Fisher exact *P*=.14).

At the 1-month follow-up, 80.3% (285/355) of the participants did not fail any attention checks, 13.2% (47/355) failed 1 check, 6.2% (22/355) failed 2 checks, and 0.3% (1/355) failed 3 checks. No one failed 4 attention checks. Similar to the baseline, recruitment type was significantly associated with data quality for the 1-month follow-up (*χ^2^*_1_=4.6, *P*=.03), with more psychology pool participants (55/241, 22.8%) failing attention checks than those from the general student body (15/114, 13.2%). In addition, failing ≥2 attention checks was significantly more prevalent among the psychology pool participants (20/241, 8.3%) than among those from the general student body (3/114, 2.6%; *χ^2^*_1_=4.1, *P*=.04).

At the 3-month follow-up, 81.7% (250/306) of the participants did not fail any attention checks, 13.1% (40/306) failed 1 check, 4.6% (14/306) failed 2 checks, and 0.7% (2/306) failed 3 checks. No one failed 4 attention checks. Significant differences in data quality were also observed for the 3-month follow-up (*χ^2^*_1_=4.2, *P*=.04), with more psychology pool participants (43/199, 21.6%) failing attention checks than the participants from the general student body (13/107, 12.1%). However, failing ≥2 attention checks was not significantly different between the psychology pool participants (13/199, 6.5%) and those from the general student body (3/107, 2.8%; Fisher exact *P*=.19).

We also examined attention check failures over time. Among the individuals who completed the 1-month follow-up survey of the 33 individuals who failed ≥1 attention checks at baseline, 16 (48%) also failed ≥1 attention checks in the follow-up survey. By contrast, of the 322 individuals who did not fail an attention check at baseline, 54 (17%) failed ≥1 attention checks in the follow-up survey. This suggested that the attention check failure at baseline was associated with the attention check failure in the follow-up survey (*χ*^2^_1_=19.01, *P*<.001). Similarly, among the individuals who completed the 3-month follow-up survey of the 31 individuals who failed ≥1 attention checks at baseline, 13 (42%) also failed ≥1 attention checks in the follow-up survey. By contrast, of the 275 individuals who did not fail an attention check at baseline, 43 (16%) failed ≥1 attention checks in the follow-up survey. This suggested that the attention check failure at baseline was again associated with the failure in the follow-up survey (*χ^2^*_1_=12.9, *P*<.001).

Controlling for year in school and age, logistic regressions with recruitment type predicting any attention check failure were not significant for baseline (B=0.65; *P*=.10; exp[B]=1.91, 95% CI 0.89-4.08) or the 3-month follow-up (B=0.69; *P*=.06; exp[B]=1.99, 95% CI 0.98-4.06), but were significant for the 1-month follow-up (B=0.77; *P*=.02; exp[B]=2.16, 95% CI 1.12-4.16). Controlling for class year and age, logistic regressions with recruitment type predicting failing ≥2 attention checks were not significant for baseline (B=1.70; *P*=.11; exp[B]=5.46, 95% CI 0.69-43.12); the 1-month follow-up (B=1.23; *P*=.06; exp[B]=3.42, 95% CI 0.96-12.27); or the 3-month follow-up (B=1.27; *P*=.06; exp[B]=3.56, 95% CI 0.94-13.57).

#### Aim 2: Study Retention by Recruitment Source

Recruitment type was associated with retention at the 1-month follow-up (*χ^2^*_1_=38.5, *P*<.001), where more participants recruited from the general student body (114/127, 89.8%) completed the 1-month follow-up than the psychology pool participants (241/401, 60.1%); similarly, more participants from the general student body completed the 3-month follow-up assessment (107/127, 84.3%) than the psychology pool participants (199/401, 49.6%; *χ^2^*_1_=47.5, *P*<.001). These comparisons were to be repeated as logistic regressions, controlling for demographics that were significantly different across recruitment sources (year in school and age). However, year in school and age did not significantly predict attention check failure at any time point, nor did they predict retention for either follow-up survey. Thus, the original chi-square comparisons served as the final models.

#### Aim 3: Impact of Data Quality on Variable Associations

Correlations among key variables for study 2 (ie, typical alcohol consumption, typical eBAC, alcohol-related problems, and descriptive norms) were conducted for the full sample, those who did not fail any attention checks, those who had failed at least one attention check, and those who failed <2 attention checks (Table 5). Before the analysis, the variables were examined for extreme values (ie, outliers) and normality. In total, 2 outliers were winsorized (or reduced to less extreme values while maintaining rank) for consumption (ie, drinks per drinking day), 5 outliers were winsorized for eBAC, 3 values were winsorized for alcohol-related problems, and 2 cases were winsorized for drinking norms (ie, perceived drinks per drinking day for close friends). Normality was confirmed for all variables. Overall, the changes in correlations were small, with several relationships increasing in strength from the full sample to the participants who did not fail an attention check. When comparing the participants who did not fail any attention checks with those who failed ≥1, the patterns of differences were larger, but there were a few relationships in a direction that was not anticipated (ie, stronger correlations among those who had failed). As expected, correlations for those who failed <2 attention checks were midrange between those who did not fail any checks and those who failed at least one check.

The largest change in correlations among those who did not fail any attention checks versus those who did were compared with Fisher *z* independent sample comparison (eBAC with alcohol-related problems), finding that the correlation was significantly stronger among those who did not fail any attention checks than among those who did (*z*=1.67; *P*=.048). The differences between all other correlations were smaller in magnitude and were not significantly different across groups by attention check failure.

**Table 5:** Correlations among the key study 2 variables categorized by attention check failure^a^.

Measure			1		2		3		4
**Full sample (N=528)**									
	1. Drinks per drinking day	—^b^		—		—		—	
	2. Typical eBAC^c^	.82		—		—		—	
	3. Alcohol-related problems	.41		.42		—		—	
	4. Descriptive norms^d^	.66		.56		.33		—	
**Did not fail any attention checks (n=464)**									
	1. Drinks per drinking day	—		—		—		—	
	2. Typical eBAC	.82		—		—		—	
	3. Alcohol-related problems	.42		.44		—		—	
	4. Descriptive norms	.66		.55		.34		—	
**Failed >1 attention checks (n=64)**									
	1. Drinks per drinking day	—		—		—		—	
	2. Typical eBAC	.82		—		—		—	
	3. Alcohol-related problems	.35		*.23*		—		—	
	4. Descriptive norms	.67		.62		*.26*		—	
**Failed <2 attention checks (n=512)**									
	1. Drinks per drinking day	—		—		—		—	
	2. Typical eBAC	.82		—		—		—	
	3. Alcohol-related problems	.42		.42		—		—	
	4. Descriptive norms	.66		.56		.33		—	

^a^All correlations were significant at *P*<.001 except for those italicized.

^b^Not available.

^c^eBAC: estimated blood alcohol concentration.

^d^Descriptive norms refer to perceived consumption (drinks per drinking day) for close friends.

#### Aim 4: Impact of Data Quality on Internal Consistency

The differences in Cronbach α for alcohol-related problems were negligible across the full sample (α=.918), omitting those who failed ≥2 attention checks (α=.917) or those who failed any attention checks (α=.917). Differences in McDonald omega was also negligible across the full sample (α=.921), omitting those who failed ≥2 attention checks (α=.920) or those who failed any attention checks (α=.920).

#### Aim 5: Demographics by Attention Check Failure

A series of chi-square analyses revealed that failing any attention checks versus none of the attention checks was not significantly associated with gender (*χ^2^*_1_=0.3, *P*=.57), ethnicity (*χ^2^*_1_=0.7, *P*=.40), year in school (*χ^2^*_5_=4.3, *P*=.51), or age (t_526_=0.38; *P*=.71). Although it was not associated with the endorsement of some racial identities (ie, identifying as Asian: *χ^2^*_1_=0.3, *P*=.60 or Native American: *χ^2^*_1_=0.3, *P*=.62), it was significantly associated with identifying as Black or African American (*χ^2^*_1_=6.6, *P*=.01), and a trend was present for identifying as White (*χ^2^*_1_=3.4, *P*=.07), although it failed to reach significance. A similar pattern was observed in study 1, where more participants who identified as Black or African American failed at least one attention check (43/277, 15.5%) than those who did not identify as Black (20/244, 8.2%), whereas fewer participants who identified as White failed attention checks (19/213, 8.9%) than those who did not identify as White (44/308, 14.3%). When comparing failing ≥2 attention checks with failing <2 attention checks, there were no significant associations between attention check failure and the demographic variables.

## Discussion

### Overview

An examination of whether data quality varies across recruitment sources (aim 1) revealed that a greater proportion of college student participants recruited through the psychology pool failed attention checks than that of those recruited through general emailed announcements, suggesting poorer data quality through satisficing. An examination of whether retention varies across recruitment sources (aim 2) revealed that the psychology pool was also associated with worse compliance via lower retention rates for the web-based follow-up surveys at 1 month and 3 months after baseline (study 2 only). For the examination of the impact of data quality on study variable associations (aim 3), there was no clear pattern of differences when comparing the strength of correlations between participants who did not fail any attention checks and those who did. The direction of the significant effect was consistent with our hypothesis for study 2 (ie, a stronger correlation was found among those who did not fail any attention checks) but was contrary to what was hypothesized for the 2 significant findings of study 1 (ie, stronger correlations were found among those who failed at least one attention check). Study 1 had 2 additional findings that were consistent with our hypothesis but did not reach significance. As for the impact of data quality on measures of internal consistency (aim 4), the impact of omitting those who failed attention checks was negligible on measures of internal consistency. Finally, when examining whether the demographic qualities of participants significantly varied across those who failed attention checks versus those who did not (aim 5), attention check failure was significantly greater among those who identified as Black or African American (both studies) and significantly lower among those who identified as White (study 1 only). It was not significantly associated with other racial identities, ethnicity, gender, age, year in school, or employment status.

Studies 1 and 2 were consistent in their findings that attention check failure rates were lower among the students recruited via general emailed announcements than among the psychology pool participants, suggesting better data quality (aim 1). This was true for both a remote, web-based, cross-sectional survey focused on college stressors and mental health (study 1) and an in-person longitudinal design examining an intervention for college drinking (study 2). However, the difference in rates was greater for the completely remote web-based study protocol (study 1: 19.3% vs 10.1% for failing any attention checks) than for the in-person baseline protocol (study 2: 13.5% vs 7.1%) for failing any attention checks, and rates were also generally higher for the web-based protocol. This finding became nonsignificant for study 2, as the sample was split into a smaller, more unbalanced proportion for examinations of failing ≥2 attention checks. Ward and Pond [44] found that having a researcher present via virtual meeting reduced careless responses by 2.13%, so having a researcher present for the in-person protocol at baseline may have reduced satisficing among participants who would otherwise have satisficed on the web (ie, changing the behavior of those enrolled in the study). In addition, the on-site protocol required that students sign up for a specific time slot and show up at a particular location on campus, requiring greater commitment. This may reflect greater motivation to participate (ie, impacting those who enrolled in the study), which has been linked to reduced satisficing among college students in prior work [14] and is consistent with the suppositions by Krosnick [16]. Thus, in-person protocols with specific sessions and researchers present may result in higher data quality through both who enrolls (self-selection of only those with greater motivation to participate) and through the protocol impacting the behavior of those enrolled (increasing motivation).

### Push-in Versus Pull-out Recruitment Approaches

Antoun et al [31] noted that pull-in recruitment sources were more efficient (ie, faster rate of enrollment and lower cost) than push-out recruitment sources. This was true for study 2, a longitudinal study with an in-person baseline session. Enrollment was much higher using the psychology pool (n=401) than the general student body contacted via emailed announcements (n=127). Similarly, the psychology pool cost is lower (using research credits as compensation rather than monetary payments). However, the findings were contradictory for study 1, which yielded lower enrollment using the psychology pool (n=127) than the emailed announcements to the general student body (n=257). Both recruitment methods were relatively low cost, with participants from the general student body compensated only with entry into one of a handful of raffles for relatively low-cost gift cards.

The finding that satisficing was greater in the pull-in recruitment source (the psychology pool) than the push-out recruitment source (emailed general announcements) was contrary to the findings of Antoun et al [31]. In total, 3 studies comparing pull-in versus push-out recruitment focused on recruitment not specific to the college population, where participant presence in the pool or panel was completely through self-selection (eg, MTurk and Qualtrics or Dynata panels) [31-33]. These individuals joined the panel specifically to participate in research and earn money. By contrast, the pull-in source for this study included students enrolled in psychology courses who could participate in research studies for course credit (either as extra credit or as part of the requirements for the class). Although they could participate in research to earn a reward, and this is the sole purpose of the panel, their existence in the pool or panel was determined through course enrollment. This could suggest that their presence in the panel was less voluntary. However, equivalent credit could be earned through article critiques rather than study participation, making study participation fully voluntary. This could suggest that which recruitment method is best depends on whether the source is college specific or general sources. One study we are aware of has compared satisficing across a general pull-in source (MTurk for US $0.50) versus a college-specific pull-in source (a psychology pool for course credit), where they operationalized satisficing using nondifferentiation (ie, selecting the same response option for all items within a scale) [45]. They found the MTurk sample engaged in more satisficing than the college psychology pool. Given that both are considered pull-in methods, it may be that compensation structure (money vs course credit) was driving this difference, with the participants completing the survey for course credit providing better-quality data. Conversely, in our study comparing 2 college sources, we found that financial compensation was associated with lower rates of satisficing, whether these payments were larger and guaranteed (US $20 for baseline in study 1) or based on chance (raffles for study 2). In particular, the pull-in approach used in this study (a psychology student participation pool at a single institution) is widely used, and results to other psychology pools are highly generalizable. However, many pull-in approaches (eg, Amazon MTurk) contain participant panels of individuals from across the country and often the globe. This makes the findings of this study less generalizable to pull-in approaches more broadly. With no robust findings across studies regarding push-in versus pull-out methods or financial compensation versus course credit, it appears that there is no guaranteed method to minimize satisficing, making its detection critically important. Attention is a prerequisite for receiving the treatment in most survey experiments, and attention checks effectively reveal who receives the treatment and who does not, such as when Berinsky et al [11] found large condition effects among those who passed the attention screener and no condition effects among those who failed. Detecting and eliminating satisficing is critical for researchers conducting studies examining treatments for psychological health.

### Longitudinal Research

The same recruitment source (announcements emailed to the general student body) provided both greater study retention (aim 2) and higher data quality (aim 1); thus, longitudinal researchers can choose a recruitment method that optimizes study compliance for both minimizing satisficing and promoting retention. It is worth noting that retention may have been better for the students who participated through student announcements because their compensation for follow-up surveys was consistent with their compensation for baseline (financial), unlike the psychology pool (course credit). Attrition for longitudinal psychological treatment studies is particularly critical, as meta-analyses have shown dropout rates of 24% to 35% for smartphone-delivered mental health interventions [46], 26% for cognitive behavioral therapy [47], 21% for eating disorder e-treatments [20], and 25% for individual college drinking interventions [48], among others. Researchers striving to minimize satisficing in their clinical trials must still try to optimize retention, and choosing an appropriate recruitment method may help with both concerns.

### Satisficing Impact on Study Findings

Contrary to our expectations, correlations did not show consistent strengthening of study variable associations or effects across the 2 studies (aim 3). Select correlations did change significantly in both studies, but the effects went in both directions (sometimes stronger and sometimes weaker). The strengthening of some correlations is consistent with multiple prior studies finding stronger effects after screening out participants who satisficed [1,11,12]. However, falsely inflated values in the full sample were similar to those reported by Huang et al [13], still pointing to a bias introduced by including these participants. Moreover, Credé [10] noted that whether random responding inflates or deflates the true value of correlations may be influenced by whether the measures examined naturally peak around the lower end of the response option continuum (such as with suicide ideation, psychopathy, and depression) versus around the higher end of the continuum (such as with self-esteem and altruistic behavior) as well as by whether the correlation among those not satisficing is positive or negative, suggesting that both inflated and deflated correlations can be expected with satisficing. Moreover, how participants are carelessly responding may influence the direction of bias. King et al [49] found that when data are not skewed, uniformly responding (ie, each response option has an equal chance of being selected) falsely deflates estimates, whereas long-string responding (ie, selecting the same response option for many items in a row) falsely inflates estimates. Thus, our findings demonstrating correlations changing in both directions support the notion that screening out participants who are satisficing does impact study findings, potentially reducing bias.

Also contrary to our expectations, measures of internal consistency were not stronger after dropping participants who failed attention checks (aim 4). The differences were negligible across the 2 studies. If satisficing by participants adds noise to the data set, researchers might expect it to add measurement error as well. Oppenheimer et al [1] found that internal consistency was reduced among those failing an instructional manipulation for a measure containing reverse-scored items, but these findings were not replicated in this study. However, only 1 measure in this study (the Penn State Worry Questionnaire) [36] contained reverse-scored items, which might be more sensitive to satisficing.

### Satisficing Detection Decisions (Number of Failures; Dropping vs Feedback)

In this study, examinations were repeated for multiple cutoffs for satisficing (ie, failing any attention checks vs failing a larger number of attention checks such as 2 or 3). A zero-tolerance approach for identifying satisficing, excluding participants who had ≥1 incorrect responses to attention checks, is consistent with what is most commonly reported by researchers [9]. Although a recent examination revealed that the zero-tolerance approach can result in excluding more participants, in particular those who do not demonstrate satisficing on other indices, it is the most common way of screening participants for data quality [9]. This study did not reveal major differences in the pattern of findings across zero tolerance versus basing the cutoff on a larger number, suggesting that there may be some flexibility in which approach a researcher might choose.

One concern raised by prior researchers is whether screening out satisficing participants could introduce a different source of bias, namely reducing the demographic diversity of the sample [11,14]. This study found that attention check failure was not significantly associated with ethnicity, gender, age, year in school, or employment status, indicating that bias is not introduced for these dimensions. However, the participants who identified as Black or African American were more likely to fail at least one attention check, suggesting that screening out participants could introduce a concern relating to reducing the diversity of the sample. Researchers should be thoughtful regarding recruitment strategies to access larger numbers of participants who could be lost to this screening process so that the final sample still has a substantial representation of this group, as in this study.

In addition to screening out those who fail attention checks, another possible approach that would allow researchers to retain everyone in the sample is to use live feedback to inform participants that the researchers have noticed that they are not paying attention and ask them to read the items carefully. Prompting respondents who completed survey items very quickly to note that this was likely too fast to respond accurately and asking whether they want to reconsider their answers led to reduced satisficing and more accurate responses [50]. Similarly, providing feedback when someone fails an attention check can increase measurement quality [51]. King et al [49] noted that almost no published research in the addiction literature reports screened their data for satisficing. It may be that for research that heavily invests resources in obtaining each data point (such as with longitudinal research and clinical trials, common in the addiction field), throwing out cases results in heavy resource loss, and researchers may be more motivated to try to detect and eliminate satisficing as it occurs. Berinsky et al [11] used different strategies to improve attention during data collection, including warning participants that their data would be monitored before beginning the survey, pairing this warning with a message thanking the participants for their time and careful attention, and providing live feedback (ie, “There was a problem with your response. Please try again”). All 3 approaches resulted in higher rates of passing the attention check items. However, these approaches did not result in reduced noise or bias in associations among study variables or larger treatment effects. It may be that the framing of these messages matter. A systematic review of studies examining prompts in health promotion or health behavior interventions found that messages were more effective if they were tailored with a personal touch [52]. Similarly, a review of retention in panel studies emphasized that explaining the importance of the project and the contributions of the participants is key to engaging participants and promoting good study retention [53]. The same approaches can be used to promote good data quality. Accentuating the purpose and importance of the study, how the participants are helping, and that their responses are of great value to the researchers may have an effect on not just passing attention checks but also actually increasing attention and minimizing noise. Pairing this warm introduction to the fact that the responses will be monitored with a similarly framed live feedback message when attention checks are failed (eg, “Your answer for this question is not correct. Your contributions to our research are extremely valuable. Please be sure to read questions thoroughly and answer carefully”) may have more of an impact on data quality.

### Recommendations

To promote data quality and minimize bias, we have several recommendations for researchers. (1) Use attention checks to detect satisficing. Failure of attention checks was prevalent in both studies across both recruitment sources, suggesting that satisficing is occurring among college students regardless of the study design or recruitment method. Moreover, findings changed after screening out those who failed at least one attention check, suggesting that ignoring this phenomenon could introduce bias into study conclusions. Attention checks can help researchers identify who is providing higher quality data. (2) Carefully consider the recruitment source. Although using psychology pools can cost less and be more efficient (as in study 2, a longitudinal study with an in-person baseline session) and potentially be more convenient, recruiting using broader methods to reach students may result in a better-quality sample (ie, lower satisficing and greater retention). Moreover, the broader recruitment source was more efficient in study 1 (remote and cross-sectional), suggesting that researchers may want to consider their study design in making this determination. When possible, researchers might use multiple recruitment sources to diversify their samples. (3) Weigh the benefits of screening out the participants who fail attention checks (demonstrated to reduced bias in study findings) versus including live feedback (very limited research on this approach). Related to this, (4) consider whether screening out participants could reduce demographic diversity. It could be problematic to increase internal validity to the detriment of external validity. If researchers intend to use attention checks for screening purposes, then they might oversample from populations more likely to be screened out (if possible). Alternatively, letting participants know that their responses will be monitored for data quality and providing live feedback could minimize attention check failure. For treatment studies or other longitudinal studies where tossing cases is problematic, live feedback may be a better option. Finally, (5) researchers interested in minimizing satisficing rather than detecting and removing the data from these participants might consider holding time-specific sessions with a researcher present (in person or on the web).

### Limitations

This investigation was a 2-study examination using different study designs (cross-sectional web-based survey vs in-person baseline for a longitudinal randomized controlled trial) and different domains of inquiry (mental health vs drinking behaviors) to maximize the external validity and relevance for other psychological health researchers. However, several limitations should be noted. First, attention checks were the only indicators of data quality used. More robust approaches have included additional indicators, such as psychometric antonyms or synonyms (ie, within-person correlations of similar items), LongStrings (ie, length of response patterns with the same value), Mahalanobis distance values (ie, multivariate outliers for similar items), and self-report items of attention and effort [8,34]. The main advantages of this approach are ease of use, nonrequirement of specialized data management skills, and speed of the screening process. However, researchers may consider using an error-balancing approach that takes multiple indices into account, particularly if working with smaller data sets of specialized populations that are harder to access, where keeping more cases is much more critical.

Another limitation of this examination was that the recruitment sources were limited to single-site data collection methods at 1 institution in the United States. Amazon MTurk is another approach that researchers can use to access the student population more broadly, potentially increasing the demographic diversity of the study samples while maintaining high data quality, including lower rates of satisficing [54,55]. Other web-based approaches, not limited to a single site, could include advertisements on Facebook, Craigslist, etc.

This may increase the demographic and geographic diversity of the sample [54], although the confirmation of student status may be harder. In addition, these pull-in methods may result in a sample with lower income that engages in greater risky behaviors [56] if such qualities are relevant to the research questions being examined. Moreover, although the samples used in this study had a strong representation of Black or African American and White racial identities, other identities were not as well represented. In particular, aim 5 had low sample sizes for some examinations. Although study 2 used a protocol that allowed us to identify all the participants and prohibited repeat sign-ups, study 1’s fully web-based protocol did not. The psychology pool participants likely also saw the survey via university-wide announcements, although the recruitment materials requested that students complete the survey only once. Unfortunately, the nature of the system used for the psychology pool uses only anonymous identifiers to issue research credits in the system, so we could not verify for ourselves that the psychology pool participants did not also complete the survey via university announcements.

Finally, although we focused on recruitment sources to label the differences between these 2 groups, compensation was also different. Students in the psychology pool were compensated with research credits that could be applied to their course grades. Students in the emailed announcement group were compensated monetarily (with a raffle entry in study 1 and direct payments in study 2). We believe that this is consistent with most studies using these recruitment sources and feel that compensation is part of these approaches. What is notable is that the pattern of reduced satisficing in the emailed announcement group was true even when the compensation was weak (raffle entry) rather than strong (direct monetary payments), suggesting that the strength of compensation is not driving the effect.

### Conclusions

This investigation examined participant compliance (ie, data quality and retention) by recruitment source across 2 studies of college students with varying design protocols (study 1: a fully remote, cross-sectional design examining college stressors and psychological health; study 2: a longitudinal design with an in-person baseline session that examined an intervention targeting college drinking). For both studies, the participants were recruited from (1) a psychology student participation pool, receiving research credit in psychology courses as compensation, and (2) the general student body via emailed announcements, receiving either a raffle entry (study 1) or monetary compensation (study 2). The examination revealed that a greater proportion of college student participants recruited through the psychology pool failed attention checks than that of those recruited through general emailed announcements, suggesting poorer data quality through satisficing in both studies. Moreover, the psychology pool was also associated with worse compliance via lower retention rates in the web-based follow-up surveys at 1 month and 3 months after baseline (study 2 only). After screening out those who failed at least one attention check, some correlations among the study variables were strengthened (potentially due to reducing noise), some were weakened, and some were fairly similar; this mixed pattern potentially points to a bias introduced by including these participants. Finally, attention check failure was not significantly associated with most demographic characteristics (ethnicity, gender, age, year in school, employment status, and select racial identities) but was greater among those who identified as Black or African American (both studies) and significantly lower among those who identified as White (study 1 only). Investigators focused on student research should carefully consider recruitment in their study design and include attention checks or other means of detecting poor quality data. Satisficing was detected across both sources, although it was worse in the psychology pool than in the general student body. Researchers should carefully consider how the study design could promote engagement (eg, live sessions with a researcher), weigh screening participants versus providing live feedback, and consider oversampling demographics that are more likely to be screened out, if possible.

## Abbreviations

eBAC: estimated blood alcohol concentration
MTurk: Mechanical Turk

## Data Availability

Data will be available from the principal investigator upon reasonable request.
